# One year follow up after implementation of a screening-package for sepsis in an emergency department

**DOI:** 10.1186/1757-7241-20-S2-P35

**Published:** 2012-04-16

**Authors:** Morten Plambech, Andrew Isak Lurie, Helle Lerche Ipsen

**Affiliations:** 1 Department of emergency medicine, Nykøbing Falster Hospital, Denmark

## Background

In 2009, Nykøbing Falster Hospital’s emergency department implemented guidelines for sepsis treatment. An education program for doctors and nurses was established and decision and therapy supporting material was made available. Key persons ensured correct implementation of the guidelines in a clinical setting. Audits were undertaken throughout the implementation period to ensure implementation. This study examines the degree of adherence to sepsis guidelines, one year after implementation.

## Methods

Study design was an interventional cohort study, utilizing the break through series and the PDSA model. Patients admitted to the emergency department were screened for sepsis during weeklong periods. Patients over 15 years of age, with verified infection and at least two SIRS criteria, were included in the study.

Structured education was conducted for personnel. Electronically accessible guidelines, posters with diagnostic and treatment algorithms, pocket references, and checklists were made available. Key nurses and doctors encouraged compliance.

Journal audits were completed before implementation (baseline), after six months (1 audit) and after one year (2 audit). Degree of compliance of six factors in sepsis guidelines (lactate measurement, oxygen and fluid treatment, timely antibiotic treatment and blood culturing, and planning treatment monitoring) was recorded.

## Results

Compliance to 3-5 of the sepsis guideline’s 6 elements increased from 37.0% at baseline (N=27) to 65.5 % at 1 audit (N=29), and decreased from 65.5% at 1 audit to 47.9% at 2 audit (N=48).

## Conclusion

It was not possible to ensure compliance to sepsis guidelines during the intervention period. Increase in compliance from baseline to 1 audit indicates that the educational program during the first 6 months had a positive effect. Subsequent decrease of compliance from 1 audit to 2 audit, is likely due to frequent rotation of doctors employed in the department. Nurses, who do not change departments as frequently, are therefore vital for establishing continuity in sepsis diagnoses and treatment. Frequent quality control and education is necessary for the effective introduction and continued effectiveness of new guidelines.

